# Multiple Functions for ORF75c in Murid Herpesvirus-4 Infection

**DOI:** 10.1371/journal.pone.0002781

**Published:** 2008-07-23

**Authors:** Miguel Gaspar, Michael B. Gill, Jens-Bernhard Lösing, Janet S. May, Philip G. Stevenson

**Affiliations:** Division of Virology, Department of Pathology, University of Cambridge, Cambridge, United Kingdom; Karolinska Institutet, Sweden

## Abstract

All gamma-herpesviruses encode at least one homolog of the cellular enzyme formyl-glycineamide-phosphoribosyl-amidotransferase. Murid herpesvirus-4 (MuHV-4) encodes 3 (ORFs 75a, 75b and 75c), suggesting that at least some copies have acquired new functions. Here we show that the corresponding proteins are all present in virions and localize to infected cell nuclei. Despite these common features, ORFs 75a and 75b did not substitute functionally for a lack of ORF75c, as ORF75c virus knockouts were severely impaired for lytic replication *in vitro* and for host colonization *in vivo*. They showed 2 defects: incoming capsids failed to migrate to the nuclear margin following membrane fusion, and genomes that did reach the nucleus failed to initiate normal gene expression. The latter defect was associated with a failure of in-coming virions to disassemble PML bodies. The capsid transport deficit seemed to be functionally more important, since ORF75c^−^ MuHV-4 infected both PML^+^ and PML^−^ cells poorly. The original host enzyme has therefore evolved into a set of distinct and multi-functional viral tegument proteins. One important function is moving incoming capsids to the nuclear margin for viral genome delivery.

## Introduction

Enzymes of DNA metabolism feature prominently among the host genes captured by herpesviruses. An increased capacity for nucleoside processing presumably once conferred on the capturing virus a selective advantage. However, evolutionary pressures change, for example as additional genes are acquired, allowing established protein folds to be put to other uses. For example, the cytomegalovirus ribonucleotide reductase homolog no longer functions as such [1] and host dCTPase homologs captured by gamma-herpesviruses are no longer dCTPases [2]. Thus, DNA sequence comparisons of herpesvirus genes can tell us about the past, but are not always informative about the present.

All sequenced gamma-herpesviruses share a homolog of the cellular formyl-glycineamide-phosphoribosyl-amidotransferase (FGARAT or FGAM synthase), which catalyzes the fourth of ten steps in *de novo* purine biosynthesis [3]. The viral homologs are more closely related to each-other than any is to the cellular gene, implying a single capture event early in gamma-herpesvirus evolution. Whether the captured gene has retained FGARAT activity is unknown. An interesting feature of rhadinovirus FGARATs is frequent gene duplication. For example, Bovine Herpesvirus-4 [4] and Ovine Herpesvirus-2 [5] both have 2 copies, and Murid Herpesvirus-4 (MuHV-4) has 3: ORFs 75a, 75b and 75c [6]. Analysis of Epstein-Barr virus lacking its (single) ORF75 homolog, BNRF1, found no defect in DNA replication after reactivation, but a 20-fold reduction in B cell transformation by the progeny virions [7]. Electron microscopy and antigen presentation assays suggested that these virions still reached endosomes. Thus, rather than functioning in viral DNA replication, BNRF1 appeared to function late in virion entry.

One approach to defining viral gene functions has been genome-wide screening. A preliminary analysis of MuHV-4 random insertion mutants [8] identified ORF75c but not ORFs 75a or 75b as essential for *in vitro* lytic replication. These data supported the idea of functional divergence. However, what these functions might be was not addressed. Analyzing MuHV-4 lytic transcripts by microarray hybridization [9]–[11] has been of limited use for ORFs 75a/b/c, because latency associated ORF73 mRNAs splice across them [12]. ORF-specific microarray probes consequently fail to distinguish dedicated ORF75a/b/c mRNAs from incompletely spliced ORF73 mRNAs. This may be why ORF50 over-expression, which down-regulates ORF73 transcription, also appears to down-regulate ORFs 75a/b/c [13].

A mass spectrometry-based analysis of MuHV-4 virions [14] identified ORF75c but not 75a or 75b. In looking for glycosaminoglycan binding, we immunoprecipitated both ORF75c and ORF75b from virions with heparin agarose [15]. The significance of this heparin binding is unclear, but the recovery of ORF75b and ORF75c argued that both are virion proteins. The KSHV ORF75 and EBV BNRF1 have also been found in virions [16], [17]. These data again suggest that the FGAGRAT homologs have acquired other functions, as promoting DNA replication is classically a function of herpesvirus early gene products rather than the virion tegument.

Genome-wide screens notwithstanding, basic facts such as the distribution of viral FGARAT homologs in infected cells remain unknown. Here we have analyzed the MuHV-4 ORFs 75a, 75b and 75c using monoclonal antibodies. We find that all 3 proteins are present in virions and that at least ORFs 75b and 75c accumulate in the nucleus after membrane fusion, even when new protein synthesis is blocked. None of the FGARAT homologs appeared to retain significant FGARAT activity. We show further that ORF75c-deficient MuHV-4 remains capable of lytic replication, although it was severely attenuated relative to the wild-type. This reflected mainly a defect in capsid transport from the site of membrane fusion in late endosomes to the nuclear margin. ORF75c also disassembled PML bodies after viral entry. Thus, the original host enzyme has evolved into a set of functionally distinct virion tegument proteins.

## Materials and Methods

### Mice

Female C57BL/6 mice were purchased from Harlan U.K. Ltd. (Bicester, U.K.), housed in the Cambridge University Department of Pathology, and infected intranasally with MuHV-4 when 6–8 weeks old. Animal welfare conformed to the UK Animal Health Act of 1981 (Home Office Project Licence 80/1992).

### Cell lines

BHK-21 fibroblasts, 293T cells, HeLa cells, human foreskin fibroblasts stably transfected with a PML-specific or a control siRNA [18], CHO cells and the FGARAT-deficient mutant CHO-AdeB [19], NIH-3T3-fibroblasts and the cre recombinase-expressing derivative 3T3-CRE [20] were grown in Dulbecco's modified Eagle medium (Invitrogen, Paisley, U.K.) supplemented with 2 mM glutamine, 100 U/ml penicillin, 100 µg/ml streptomycin and 10% fetal calf serum (PAA laboratories, Linz, Austria). To select FGARAT^+^ cells, the fetal calf serum was dialyzed extensively to remove free purines and the medium was supplemented with 100 µM hypoxanthine (Sigma-Aldrich, Poole, U.K.). Cells were transfected where indicated using Fugene-6 (Roche Diagnostics, Ltd., Lewes, U.K.).

### Plasmids and viral mutagenesis

ORFs 75a (genomic co-ordinates 117904-114029 of Genbank accession number U97553), 75b (113901-110074) and 75c (109999-106067) [7] were amplified by PCR (Phusion DNA polymerase, New England Biolabs, Hitchin, U.K.) using primers that added a 5′ *Mfe*I site and a 3′ *Sal*I site to each coding sequence. The PCR products were cloned into the *Eco*RI/*Xho*I sites of pcDNA3 (Invitrogen). To disrupt ORF75c, a *Bam*HI genomic clone spanning genomic co-ordinates 107477-111869 [21] was digested with *HpaI* (109078) and dephosphorylated with Antarctic alkaline phosphatase (New England Biolabs). A self-complementary oligonucleotide encoding multiple stop codons, 5′-CTAGCTAGCTAGAATTCTAGCTAGCTAG-3′, was annealed, phosphorylated with polynucleotide kinase (New England Biolabs) and ligated into the *Hpa*I site. The mutated genomic clone was then sub-cloned as a *Bam*HI-digested fragment into the *Bam*HI restriction site of the pST76K-SR shuttle vector, and recombined into the MuHV-4 BAC (strain MHV-68) by transient RecA expression [22]. Three independent mutants were isolated (75c^−^.4, 75c^−^.7, 75c^−^.9) plus revertants of 75c^−^.4 (revertant.1) and 75c^−^.7 (revertant.2) in which the wild-type genomic sequence was recombined into the BAC in place of the inserted oligonucleotide. ORF50^−^ MuHV-4, in which genomic co-ordinates 69177-67792 are replaced by the luciferase coding sequence, was kindly provided by Dr. Stacey Efstathiou (Division of Virology) and propagated in NIH-3T3 cells with tetracycline-inducible ORF50 expression. This mutant will be described in detail elsewhere. Infectious virus was reconstituted by transfecting BAC DNA into BHK-21 cells. The loxP-flanked BAC/eGFP cassette was removed where indicated by virus passage through 3T3-CRE cells. Virus stocks were then grown in BHK-21 cells [23], passaging the cells as required until most showed cytopathic effects. This was typically 4–5 days post inoculation with 0.001 p.f.u./cell for ORF75c^+^ viruses and approximately 2 weeks post-inoculation for ORF75c^−^ viruses. Cell debris was pelleted by low-speed centrifugation (1000×*g*, 3 min) and discarded. Virions were then recovered from supernatants by high speed centrifugation (38,000×*g*, 90 min) and stored at −70°C.

### Virus titrations

Infectious virus was titered by plaque assay on BHK-21 cells [24]. 10-fold virus dilutions were incubated on cell monolayers (2 h, 37°C), then overlaid with 0.3% carboxymethylcellulose. The monolayers were fixed in 4% formaldehyde 4 days later and stained with 0.1% toluidine blue. We also assayed the infectivity of viruses retaining the loxP-flanked BAC cassette by eGFP expression from its HCMV IE1 promoter. Cells were exposed to 10-fold virus dilutions (18 h, 37°C), then trypsinized, washed and analyzed for eGFP expression by flow cytometry. The genome content of virus stocks was quantitated by real-time PCR (Rotor-Gene, Corbett Research) of the MuHV-4 M2 gene (forward primer genomic co-ordinates 4166-4188, reverse primer 4252-4228, Taqman probe 4219-4189). The viral genome content of *ex vivo* tissue samples was analyzed similarly, with adenosine phosphoribosyl transferase (APRT) [25] amplified in parallel as a cellular control (forward primer GGGGCAAAACCAAAAAAGGA, reverse primer GCTGGAATTACCGCGGCT, probe CGCAAATTACCCACTCCCGACCC).

### Viral transcript analysis

RNA was recovered from MuHV-4-infected cells and reverse transcribed (MessageSensor RT kit, Ambion, Warrington, U.K.) with a 3′ gene-specific primer, followed by real-time PCR with the same primer plus a 5′ partner. PCR products were quantitated with a gene-specific probe. We analyzed ORF73 (forward primer genomic co-ordinates 104047-104063, reverse primer 104173-104153, probe 104079-104104), ORF50 (forward primer genomic co-ordinates 66779-66795/67661-67664 spanning ORF50 exon1/exon2, reverse primer 67775-67756, probe 67698-67670) and the cellular 18S rRNA as a control (forward primer CGGCTACCACATCCAAGGAA, reverse primer TGTGTGTGGGGCCTGAGTC, probe TGCCTAAACACAAGCATCCCTACCTCAA). PCR primers and HPLC-purified Taqman probes were manufactured by TIB-Molbiol (Berlin, Germany). A standard curve for each primer set was generated by parallel amplifications of plasmid template dilutions, and the average copy number of triplicate PCR reactions for each sample calculated from this.

### Southern blotting

Viral DNA was extracted from virus stocks by alkaline lysis [24], digested with *Eco*RI, electrophoresed through 0.8% agarose in Tris acetate buffer and transferred to positively charged nylon membranes (Roche Diagnostics). A ^32^P-dCTP labelled probe (APBiotech, Little Chalfont, U.K.) was generated by random primer extension (Nonaprimer kit, Qbiogene, Bingham, U.K.) of the *Bam*HI-H genomic clone (genomic co-ordinates 107477-111869) [21]. The membranes were hybridised with the probe (65°C, 18 h), washed in 30 mM sodium chloride/3 mM sodium citrate/0.1% sodium dodecyl sulfate at 65°C, and exposed to X-ray film. Circular and linear genomes were distinguished by Gardella gel analysis [26]. Virus-infected cells (2 p.f.u./cell, 18 h) were resuspended in 15% Ficoll/100 µg/ml RNAase A and 10^6^ cells/lane loaded into a vertical agarose gel. The cells were then overlaid with an equal volume of 5% Ficoll/1% SDS/100 µg/ml proteinase K and electrophoresed to resolve linear and circular viral genomes. The DNA was transferred to nylon membranes as above and probed with a ^32^P-dCTP labelled probe corresponding to a 1.2 kb *Pst*I-restricted fragment [21] from the MuHV-4 terminal repeats.

### Monoclonal antibodies (mAbs)

All mAbs were derived from MuHV-4-infected BALB/c mice by fusing spleen cells with NS0 myeloma cells [27]. MAbs specific for ORF75a (BN-3H8), ORF75b (CS-4A1) and ORF75c (BN-6C12) are described in the results section. MAbs T3B8 (gp70, IgG_1_) [15], T2C12 (gH/gL, IgG_2a_) [28], MG-4D11 (gB, IgG_2a_) [29], MG-12B8 (ORF65 capsid component, IgG_2a_) [29], 3F7 (gN, IgG_2a_) [30], CS-4A5 (thymidine kinase) [31] and 150-7D1 (ORF17 capsid component, IgG_2a_) [29] have been described. MAb BH-6D3 (ORF25 capsid component, IgG_1_) was identified as such by mass spectrometry of a 160 kDa protein it precipitated from MuHV-4 virion lysates (Fig.S1).

### Immunofluorescence and confocal microscopy

Adherent cells (BHK-21, 293T or NIH-3T3) were washed in PBS, fixed in 4% paraformaldehyde (15 min), then permeabilized with 0.1% Triton-X100 (30 min) and blocked with 10% fetal bovine serum in PBS (60 min). MuHV-4 virion components were detected with mAbs (see above) plus Alexa488- or Alexa568-labeled anti-mouse IgG (Invitrogen, Paisley, U.K.) or Alexa488- or Alexa633- labeled anti-mouse IgG_1_ plus Alexa488- or Alexa568- labeled anti-mouse IgG_2a_. EGFP fluorescence was visualized directly. PML was detected with mAb PG-M3 (IgG_1_) (Santa Cruz Biotechnology, Santa Cruz). The cells were washed ×2 in PBS/0.1% Tween-20 after each antibody incubation and mounted in ProLong Gold anti-fade reagent with DAPI (Invitrogen). Fluorescence was visualized with an Olympus IX70 microscope plus a Retiga 2000R camera line (QImaging) or with a Leica SP2 confocal microscope.

### Metabolic labelling and immunoprecipitation

BHK-21 cells were infected with MuHV-4 (6 h, 37°C, 3 p.f.u./cell) then washed ×2 in PBS, and labelled for 2 h in cysteine/methionine-free medium with dialysed fetal calf serum plus ^35^S-labelled cysteine-methionine [32], followed by a further 40 h in the same medium plus 3% non-dialyzed fetal calf serum. Cell debris was pelleted (1000×*g*, 10 min) and virions recovered from the cleared supernatants by ultracentrifugation (20,000×*g*, 2 h). Each fraction was lysed (30 min, 4°C) in 1% Triton X-100, 50 mM TrisCl pH 7.4, 150 mM NaCl, 5 mM EDTA, 1 mM PMSF, plus Complete protease inhibitors (Roche Diagnostics). Insoluble debris was removed by centrifugation (13,000×*g*, 15 min). The supernatants were precleared with normal rabbit serum plus protein A/protein G-sepharose. Thymidine kinase, ORF75a and ORF75c were then immunoprecipitated with specific mAbs followed by protein A/protein G-sepharose. The sepharose beads were washed ×5 in lysis buffer. The precipitated proteins were then eluted and denatured by heating (95°C, 5 min) in Laemmli's buffer and resolved by SDS-PAGE. Dried gels were exposed to X-ray film.

### Immunoblotting

Virions were lysed and denatured by heating (95°C, 5 min) in Laemmli's buffer. Virion proteins were resolved by SDS-PAGE, transferred to PVDF membranes and probed with MuHV-4-specific mAbs plus horseradish peroxidase-conjugated rabbit anti-mouse IgG pAb (Dako Cytomation, Ely, U.K.), followed by ECL substrate development (APBiotech) [33]. To visualize all proteins, gels were stained with 0.15% Coomassie R-250 in 50% methanol/10% acetic acid (30 min), then destained in the same buffer without dye (1 h) and further in 5% methanol/7% acetic acid (18 h).

### Flow cytometry

BHK-21 cells were left uninfected or exposed to BAC^+^ ORF75^+^ or ORF75^−^ MuHV-4 (18 h, 37°C), then trypsinized, washed ×2 in PBS and analyzed for viral eGFP expression by flow cytometry using a FACSort (BD Biosciences, Oxford, U.K.).

### Electron microscopy

BHK-21 cells were infected with wild-type MuHV-4 (1 p.f.u./cell, 18 h) or with ORF75c^−^.4 MuHV-4 to an equivalent level of viral eGFP expression, then washed in 0.9% NaCl, fixed in 2% Glutaraldehyde/0.3% H_2_O_2_ (2 h, 4°C), washed in 0.1 M HEPES buffer pH = 7.4, and stained and embedded for transmission electron microscopy [24].

## Results

### Identification of the ORF 75a, 75b and 75c gene products

**Figure 1:**
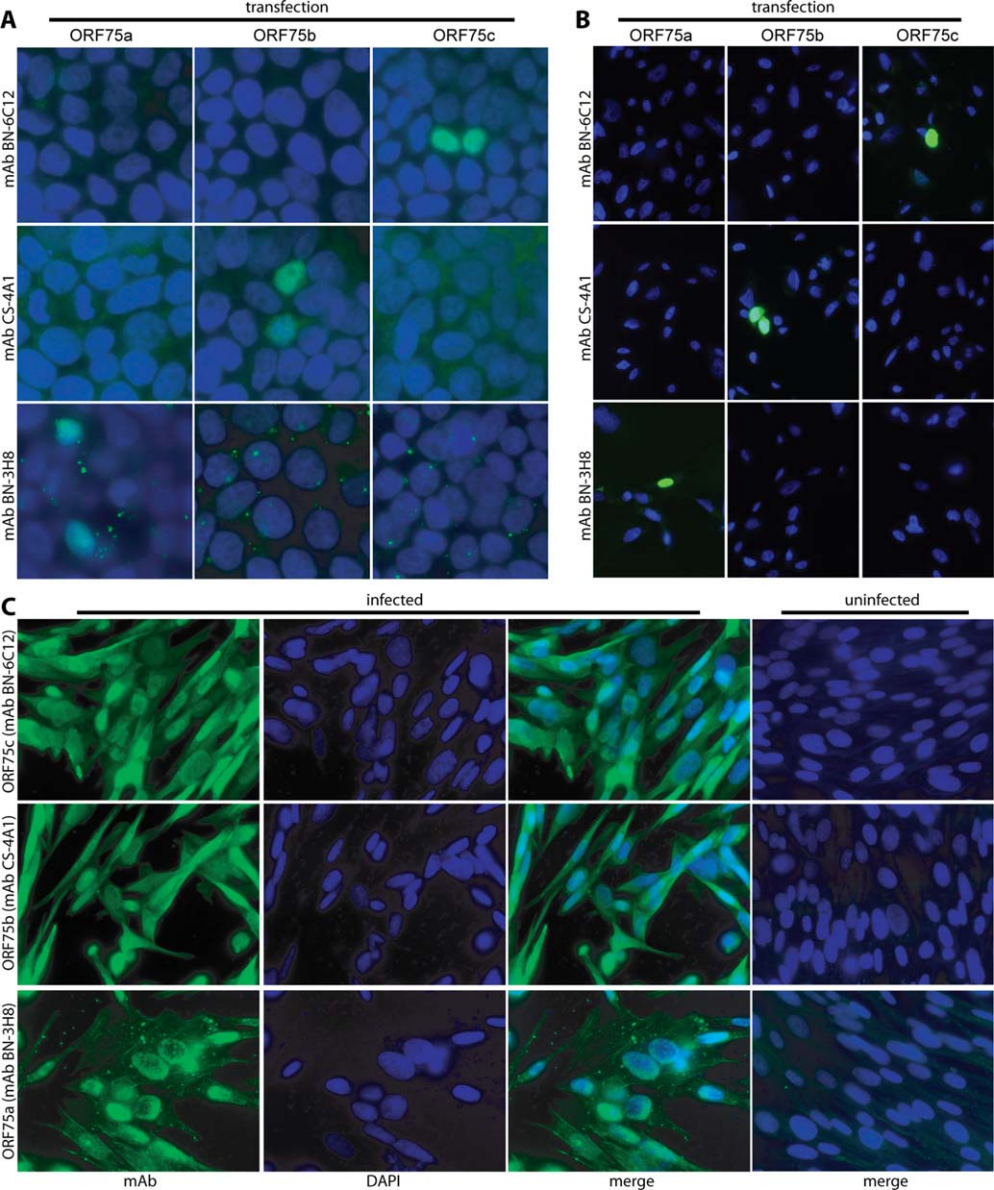
Immunostaining of ORFs 75a/b/c. A. 293T cells were transfected with a mammalian expression vector encoding ORF75a, ORF75b or ORF75c. 48 h later the cells were fixed in 4% paraformaldehyde, permeabilized with Triton-X100 and stained with mAbs derived from MuHV-4-infected mice. Positive staining is green. Nuclei were counter-stained with DAPI (blue). Each field shown is representative of at least 50 examined. B. FGARAT-deficient CHO-AdeB cells were transfected with ORF75a/b/c expression constructs as in A, then selected for 2 weeks in G418 and switched to purine-free medium with hypoxanthine for a further 3 weeks. This led to growth arrest. The cells were then switched back to normal medium and ORF75a/b/c expression tested by immunostaining as in A. C. BHK-21 cells were infected with wild-type MuHV-4 (2 p.f.u./cell, 18 h), then fixed in 4% paraformaldehyde, permeabilized with Triton-X100 and stained with ORF75a/b/c-specific mAbs. Nuclei were counterstained with DAPI. Only merged images are shown for the uninfected controls.

Our first aim was to derive antibodies capable of identifying the ORF75a, ORF75b and ORF75c gene products. To this end, B cell hybridomas were derived from MuHV-4-infected mice by fusing their spleen cells with NS0 myeloma cells. Antibodies specific for ORFs 75a, 75b or 75c were identified by using hybridoma supernatants to stain 293T cells transfected with the corresponding expression plasmids (Fig. 1A). Although none of the viral FGARAT homologs has an obvious nuclear localization signal, all localized to the nucleus after transfection.

We also stably expressed each FGARAT homolog in FGARAT-deficient CHO-AdeB cells. These showed the same nuclear localization of ORF75a/b/c (Fig. 1B). None of the viral ORFs was able to rescue these cells for growth in purine-deficient medium supplemented with hypoxanthine. Thus, both ORF75a/b/c-transfected and untransfected CHO-AdeB cells (but not normal CHO cells) underwent growth arrest in purine-deficient medium. No clones grew out of the transfected populations, and immunofluoresence established that 3 weeks of culture in purine-deficient medium had failed to enrich for ORF75a/b/c expression. By this measure, therefore, none of the ORF75 homologs retained significant FGARAT activity.

We next determined the distributions of ORF75a/b/c in MuHV-4-infected cells (Fig. 1C). Again, all 3 proteins were present in nuclei. The main difference from transfected cells was additional cytoplasmic staining. Herpesvirus secondary envelopment occurs in the trans-Golgi network, so tegument proteins generally accumulate in the cytoplasm to await packaging into virions [34]. The additional cytoplasmic ORF75a/b/c staining of infected cells was therefore consistent with the ORF75a/b/c proteins being packaged into virions.

### ORFs 75a, 75b and 75c all encode virion proteins

**Figure 2:**
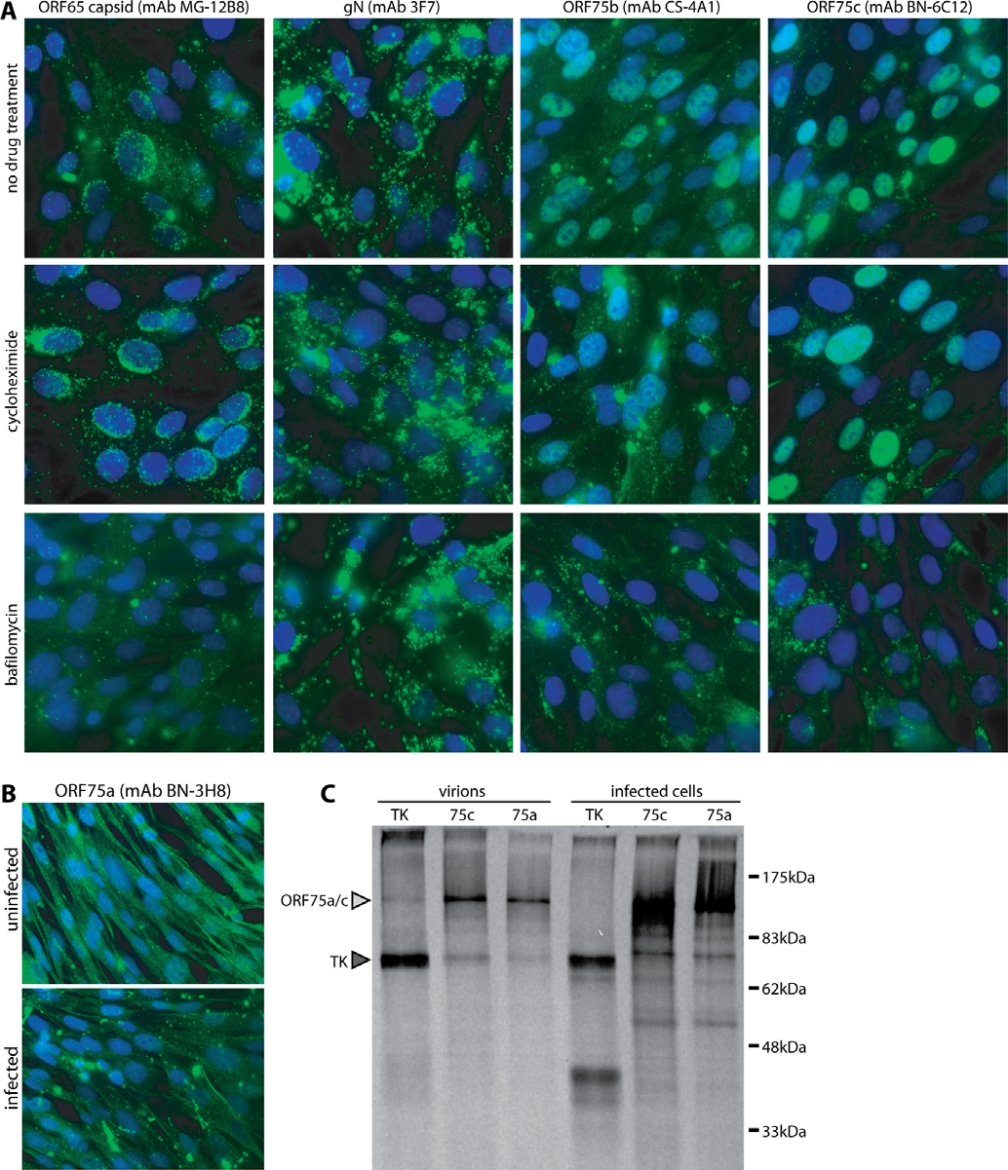
Identification of ORFs 75a/b/c as virion components. A. BHK-21 cells were incubated with wild-type MuHV-4 virions (5 p.f.u./cell, 6 h, 37°C), with or without cycloheximide (100 µg/ml) to block new protein synthesis or bafilomycin (500 nM) to block virion membrane fusion. The cells were then fixed in 4% paraformaldehyde, permeabilized with Triton X-100 and stained (green) for ORF75b, ORF75c, the ORF65 virion capsid component or glycoprotein N (gN). Nuclei were counterstained with DAPI (blue). B. BHK-21 cells were exposed to MuHV-4 virions as in A without drug treatment, then fixed, permeabilized and stained for ORF75a. At the exposure necessary to see strong positive staining, background staining of uninfected controls was also evident. C. ^35^S-cysteine/methionine-labelled, MuHV-4-infected BHK-21 cultures were separated into infected cell and virion fractions, lysed in Triton X-100 and precipitated with mAbs specific for thymidine kinase (TK), ORF75c (75c) or ORF75a (75a), plus protein A/protein G-sepharose. Immunoprecipitated proteins were separated by SDS-PAGE and visualized by autoradiography. The predicted sizes of ORF75a (142 kDa), ORF75c (146 kDa) and TK (72 kDa) are marked.

We reasoned that if ORFs 75a/b/c were virion components they would be delivered into newly infected cells, and might therefore appear in the nuclei of these cells shortly after membrane fusion, even without new protein synthesis. To test this, we exposed BHK-21 cells to wild-type MuHV-4 virions for 4 h, with or without concurrent cycloheximide treatment to block protein synthesis, then stained the cells for ORFs 75a, 75b and 75c. The cells were also stained for glycoprotein N and the ORF65 capsid component. As a further control, we treated some cells with bafilomycin to block viral membrane fusion [28] and therefore tegument protein delivery (Fig. 2A). Without drug treatment, ORF65 staining showed incoming capsids accumulating around the nuclear margin. As expected, this was dependent on membrane fusion - the MG-12B8 epitope is inaccessible on intact virions [28] - and independent of new protein synthesis. The few capsid antigens visible with bafilomycin treatment did not show a peri-nuclear localization and probably corresponded to damaged virions or infected cell debris present in the virus stock. Glycoprotein N (mAb 3F7) was accessible, and by its distribution endocytosed regardless of drug treatment.

ORFs 75b and 75c both accumulated in the nuclei of cells exposed to virions regardless of cycloheximide treatment. They therefore appeared to be virion components rather than immediate-early gene products. Neither was identifiable in the nuclei of bafilomycin-treated cells. Instead, there was staining of cytoplasmic inclusions in a similar pattern to gN. These data were consistent with ORFs 75b and 75c being endocytosed as components of virions, then homing to the nucleus when released from the virion tegument by membrane fusion.

ORF75a nuclear staining was hard to discern in newly infected cells, either because this antibody was less good for detection or because MuHV-4 virions contain less ORF75a protein than ORF75b or ORF75c. Fig. 2B makes clear that even exposure times sufficient to show background staining of uninfected cells failed to reveal convincing nuclear staining of infected cells. The main additional staining of infected cells was in cytoplasmic inclusions suggestive of endocytosed virions.

Because the detection of incoming ORF75a by immunofluorescence was weak, we confirmed its presence in virions by immunoprecipitation from virion lysates (Fig. 2C). MuHV-4-infected BHK-21 cells were labelled with ^35^S-cysteine/methionine from 6–48 h post-infection. Infected cell debris was then removed by centrifugation, cell-free virions were recovered from the cleared supernatants by ultracentrifugation, and ORF75a was immunoprecipitated from each fraction. The known virion proteins ORF75c and thymidine kinase (TK) were immunoprecipitated in parallel as controls. ORF75a was clearly recoverable from both virions and infected cells, much like ORF75c. A 70 kDa protein co-precipitated, albeit weakly, with both ORF75a and ORF75c from virions but not from infected cells. This protein was equivalent in size to TK, and the TK-specific mAb reciprocally co-precipitated a 150 kDa band from virions but not from infected cells. These data were consistent with TK and ORF75a/ORF75c associating in the virion tegument. The co-precipitation with TK of a 45 kDa band from infected cells but not from virions pointed to additional changes in protein association during virion assembly. This was not pursued further. The main conclusion of the immunoprecipitations was that ORF75a, like ORF75c, was present in virions.

### ORF75c-deficient MuHV-4 is replication-competent but highly attenuated

**Figure 3:**
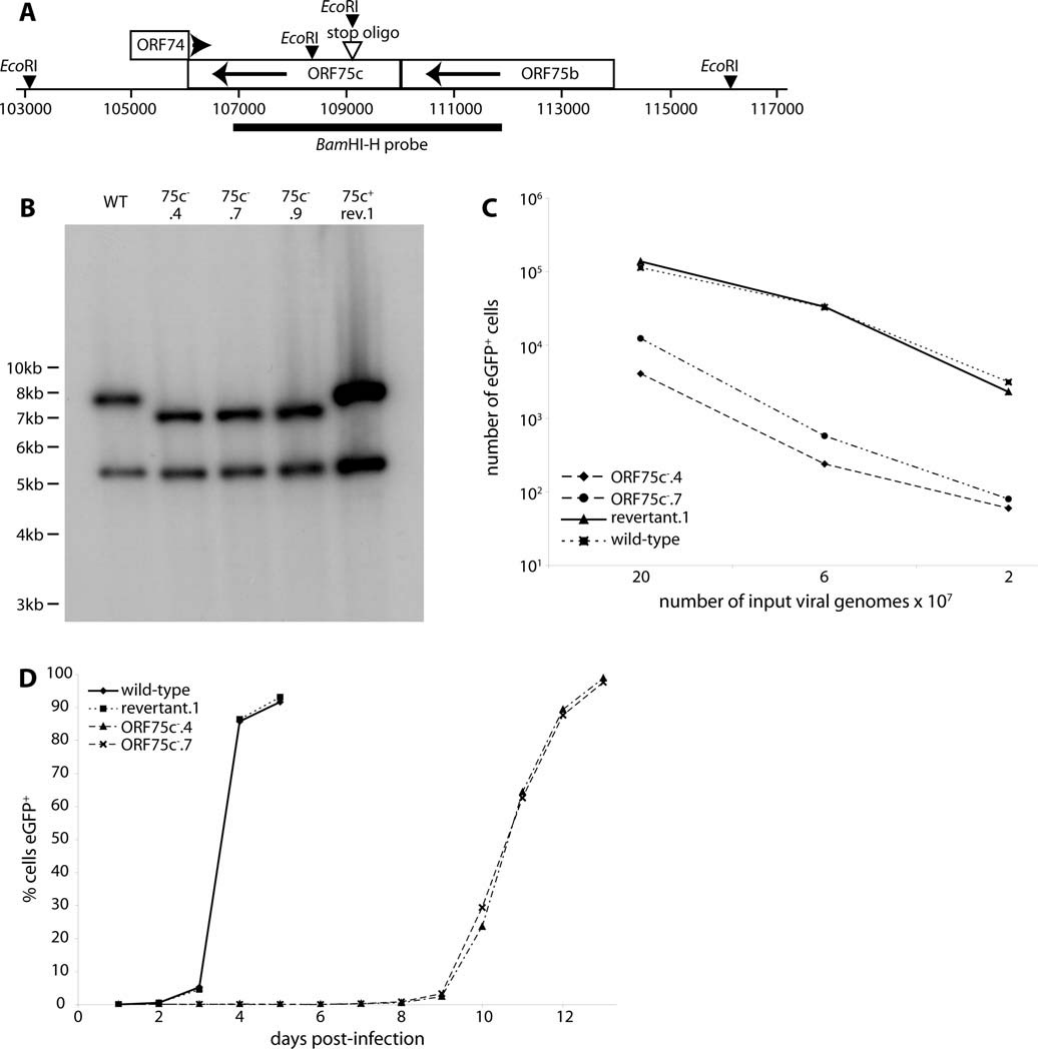
Generation of ORF75c^−^ MuHV-4 mutants. A. Schematic diagram of the ORF75c genomic locus and the introduced stop oligo mutation. B. Southern blot of viral DNA recovered from wild-type (WT), ORF75c mutant (75c^−^.4, 75c^−^.7, 75c^−^.9) and 75c^−^.4 revertant (75c^+^rev.1) virus stocks. The viral DNA was digested with *Eco*RI and probed with the labelled *Bam*HI-H genomic fragment shown in A. There is an invariant band of 5186 bp. The wild-type 7776 bp band shifts to 7048 bp in the mutant. The small mutant band of 728 bp is not visible on this gel. C. Two ORF75c^−^ mutants were compared with wild-type and revertant viruses for infectivity. The viral genome content of each stock was determined by real-time PCR, and equivalent genome numbers used to infected BHK-21 cells (18 h, 37°C). The number of infected cells was determined by flow cytometry of eGFP expression from the MuHV-4 BAC cassette. D. BHK-21 cells were infected with ORF75c^−^ (ORF75c^−^.4, ORF75c^−^.7) or ORF75c^+^ (wild-type, revertant.1) viruses at 10 genomes/cell - equivalent for the wild-type and revertant to 0.01 p.f.u./cell. The spread of infection with time was assayed by flow cytometry of viral eGFP expression.

In so far as ORFs 75a/b/c all encoded tegument proteins that localized to the nuclei of infected cells, they appeared to be quite similar. However, the viability of ORF75a and ORF75b but not ORF75c mutants [8] indicated important functional differences. As a first step in identifying what these functions might be, we generated a virus lacking ORF75c. An oligonucleotide encoding multiple stop codons was inserted close to the ORF75c N-terminus (Fig. 3A). Restriction enzyme mapping of BAC DNA (data not shown) and Southern blots of viral DNA (Fig. 3B) confirmed the expected genome changes.

The propagation of ORF75c^−^ mutants after BHK-21 cell transfection with BAC DNA was noticeably worse than that of the wild-type. ORF75c^+^ viruses all spread rapidly, whereas several cell passages were required before ORF75c^−^ viral replication outstripped cell division. ORF75c^−^ plaque formation was correspondingly poor: the cells tended to over-grow before a plaque was formed. We therefore compared ORF75c^+^ and ORF75c^−^ virus stocks primarily by viral genome content, using real-time PCR, and we assayed infectivity by eGFP expression from a Human cytomegalovirus (HCMV) IE1 promoter in the BAC cassette rather than by plaque formation. The genome∶eGFP expression ratio was 10–100-fold higher for ORF75c^−^ mutants than the wild-type, depending on the multiplicity of infection (Fig. 3C). Thus, it appeared that many more ORF75c^−^ particles were required to establish an infection.

We performed growth curves by measuring the %eGFP^+^ cells with time in BHK-21 cell populations exposed to BAC^+^ viruses at low multiplicity (Fig. 3D). This approach provided further quantitation of the ORF75c^−^ replication deficit. It also emphasized that although ORF75c^−^ mutants were attenuated, they remained replication competent. This difference from transposon screening, where ORF75c was identified as essential [8], presumably reflects that genome-wide screens can miss details such as low efficiency propagation. With single gene mutants, it is more feasible to alter *in vitro* growth conditions, such as cell density, to take account of attenuation. There was no sign that the ORF75c^−^ viruses recovered from *in vitro* growth curves replicated faster than the original inocula, and they remained ORF75c^−^ by immunofluorescence and by DNA sequence across the mutation site (data not shown).

### Normal ORF75c^−^ virion morphogenesis

**Figure 4:**
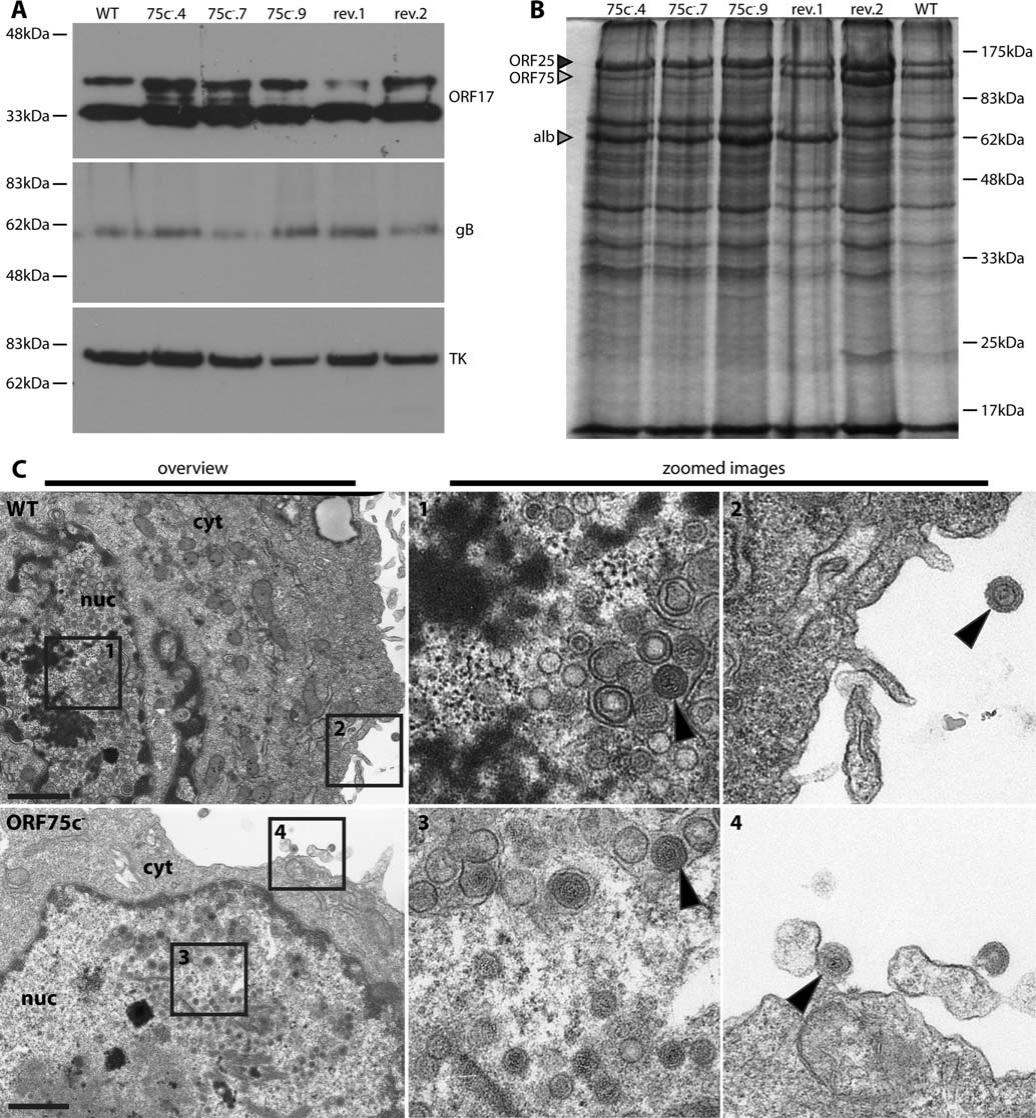
No evidence for ORF75c deficiency compromising virion morphogenesis. A. Wild-type (WT), ORF75c^−^ (75c^−^.4, 75c^−^.7, 75c^−^.9) and revertant (rev.1, rev.2) virus stocks were normalized by genome content, denatured in Laemmli's buffer and immunoblotted for the ORF17 capsid component with mAb 150-7D1, for gB (C-terminal furin cleavage product) with mAb MG-4D11, and for thymidine kinase (TK) with mAb CS-4A5. B. Virus stocks were denatured, resolved by SDS-PAGE and analyzed by Coomassie staining. The positions of bovine albumin (alb, 63 kDa), the ORF25 capsid component (160 kDa) and the ORFs 75a/b/c (150 kDa) are shown. C. BHK-21 cells were infected with wild-type (WT) MuHV-4 (1 p.f.u./cell, 18 h) or ORF75c^−^.4 (ORF75c^−^) MuHV-4 to an equivalent infection level based on viral eGFP expression, then processed for transmission electron microscopy. The scale bars show 1 µM in the overview images. nuc = nucleus, cyt = cytoplasm. The boxed regions in the overview images correspond to the zoomed images 1–4. The arrows show examples of mature virions.

Since ORF75c appeared to be a component of the MuHV-4 tegument, a possible explanation for the replication deficit of ORF75c knockouts was that they failed to assemble virions properly. However, immunoblots established that in ORF75c^+^ and ORF75c^−^ virus stocks, equivalent genome numbers corresponded to roughly equivalent amounts of virion capsid (ORF17), glycoprotein (gB) and tegument (thymidine kinase) (Fig. 4A). The only obvious abnormality on Coomassie staining of virion lysates (Fig. 4B) was a reduction in the 150 kDa band that contains ORF75c (Fig. 2C), ORF75a (Fig. 2C), ORF75b [15] and gp150 [15]. The visible reduction in protein content by ORF75c disruption alone implied that ORF75c, or a virion protein that requires it for packaging, is the major component of this band. Finally, electron microscopy showed no obvious morphological difference between ORF75c^+^ and ORF75c^−^ infected BHK-21 cells (Fig. 4C). In particular, we could readily identify morphologically normal ORF75c^−^ virions being released from infected cells.

### ORF75c^−^ virions are deficient in establishing both lytic and latent infections

**Figure 5:**
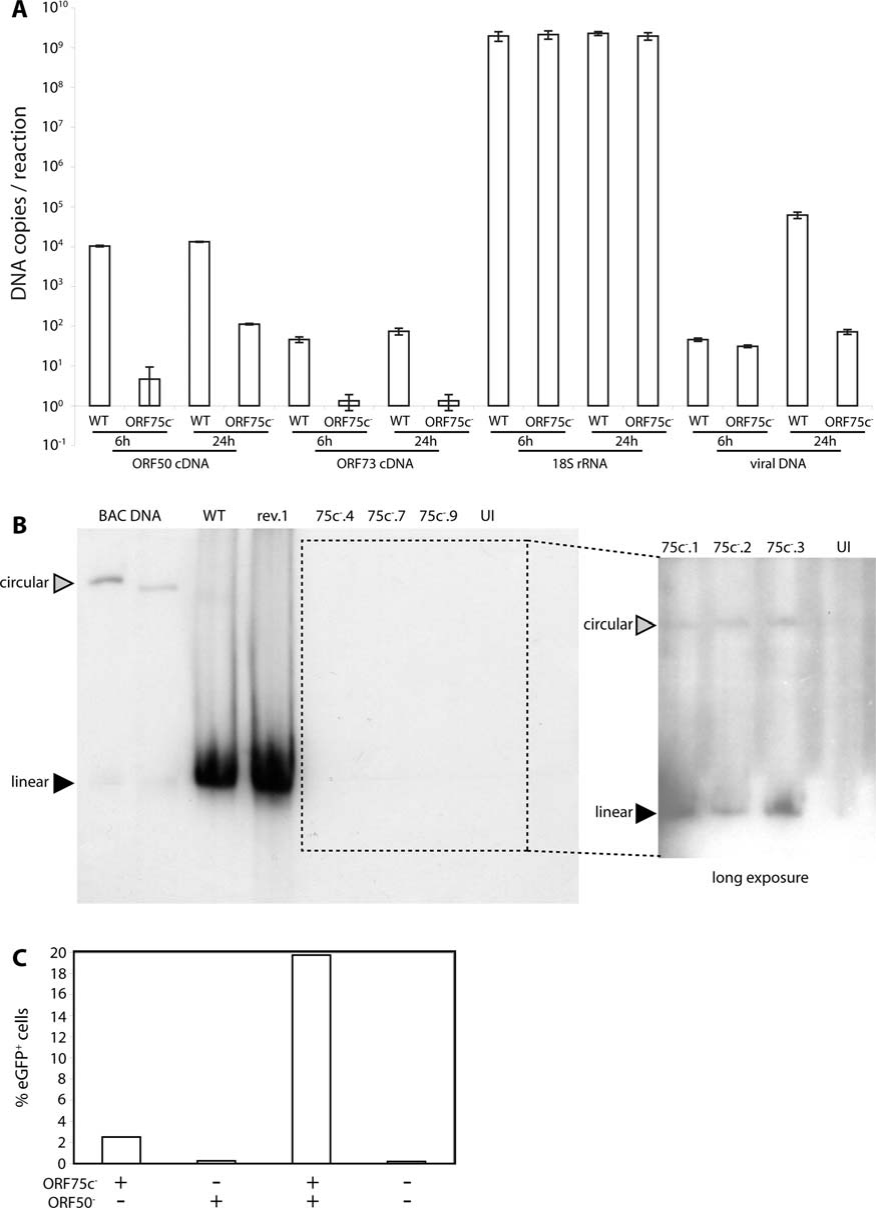
ORF75c^−^ MuHV-4 shows defective immediate early gene expression. A. BHK-21 cells were infected (1000 genomes/cell) with wild-type (WT) or ORF75c^−^.4 (ORF75c^−^) virions. DNA and RNA were recovered at 6 h and 24 h post-infection. RNA was reverse transcribed using ORF50, ORF73 and 18S rRNA-specific primers and cDNAs quantitated by real-time PCR. Controls without reverse transcriptase were all negative. Each sample was run in triplicate and mean copy numbers determined by comparison with known template dilutions. Viral genome numbers were quantitated by amplificating 100 ng DNA with MuHV-4 M2-specific primers and comparing with known template dilutions. B. BHK-21 cells were infected with ORF75c^+^ (WT, rev.1) or ORF75c^−^ (75c^−^.4, 75c^−^.7, 75c^−^.9) viruses (2 p.f.u./cell or an equivalent number of genomes), and 18 h later lysed *in situ* in agarose gels. UI = uninfected cells. Circular and linear genomes were distinguished by electrophoretic mobility and comparison with circular genomic BACs, which differ in size due to different numbers of terminal repeats. Viral genomes were identified by probing with a labelled terminal repeat fragment. The boxed area was exposed for a longer time to visualize ORF75c^−^ genomes. C. BHK-21 cells were infected with BAC^+^ORF75c^−^ MuHV-4 (1000 genomes/cell), then 24 h later super-infected with BAC^−^ORF50^−^ MuHV-4 (1000 genomes/cell), then 24 h later analyzed for BAC-based eGFP expression by flow cytometry. Each bar shows 20,000 cells. The data are from 1 of 3 equivalent experiments. The increase in eGFP expression with ORF50^−^ superinfection was highly significant (p<10^−6^ by Chi-squared test).

Since electron microscopy showed no ORF75c-dependent virion assembly defect, the limited viability of ORF75c^−^ viruses seemed likely to reflect a problem in establishing infection. Cell binding and penetration depend primarily on virion glycoproteins, but post-fusion events may depend on the tegument. One possibility suggested by the nuclear localization of incoming ORF75c (Fig. 3A) was that it might function as a lytic gene transactivator like the Herpes simplex virus VP16 [35]. If so, we would expect transcription of the lytic switch gene, ORF50, to be reduced without ORF75c, and that of latency genes such as ORF73 [12] to be relatively preserved. To test this, we exposed cells to ORF75c^+^ or ORF75c^−^ virions, then quantitated ORF50 and ORF73 mRNAs by reverse transcription-real-time PCR (Fig. 5A). When ORF75c was lacking, ORF73 transcripts were reduced by at least as much as ORF50. Thus, the infection block appeared to affect viral gene expression generally rather than just in the lytic cycle. This result was consistent with ORF75c mutants showing poor BAC cassette-based eGFP expression (Fig. 3C), since the HCMV IE1 promoter driving eGFP expression operates quite independently of endogenous MuHV-4 transcription [36], [37]. We also found no evidence that co-transfected ORF75c transactivates the ORF50 promoter in CAT assays (data not shown).

Gardella gel analysis of cells exposed to ORF75c^+^ or ORF75c^−^ virions (Fig. 5B) confirmed a lack of ORF75c^−^ viral DNA replication (Fig. 5A) - ORF75c^−^ genomes could only be visualized with long exposure times. A relatively high proportion of these genomes appeared to be circular rather than linear. Since herpesvirus genomes are linear when packaged into virions and only circularize in the nucleus [38], this result suggested that genomes still reached the nucleus without ORF75c, but failed to amplify the genome load by lytic replication. In conjunction with the earlier transcription analysis (Fig. 5A), these data suggested that ORF75c^−^ infection was blocked between genome circularization and the initiation of either lytic or latent transcription.

We tested whether the ORF75c^−^ genomes in virus-exposed cells might remain viable by super-infecting these cells 24 h later with ORF50^−^ virions (Fig. 5C). ORF50^−^ mutants are non-viable unless grown in a complementing cell line [39]. Thus in non-complementing cells they deliver tegument proteins such as ORF75c but do not initiate lytic infection. BHK-21 cells were exposed to BAC^+^ORF75c^−^ virions then super-infected with BAC^−^ORF50^−^ virions. There was significant ORF75c^−^ genome rescue as judged by BAC-based eGFP expression. Because the ORF50^−^ and ORF75c^−^ genomes could potentially recombine with complicated results, detailed analysis of the super-infections was not attempted. But it seemed clear that some otherwise non-functional ORF75c^−^ genomes could be rescued by delivering an ORF75c^+^ virion tegument.

### ORF75c destroys PML bodies, although this does not explain the ORF75c^−^ replication deficit

**Figure 6:**
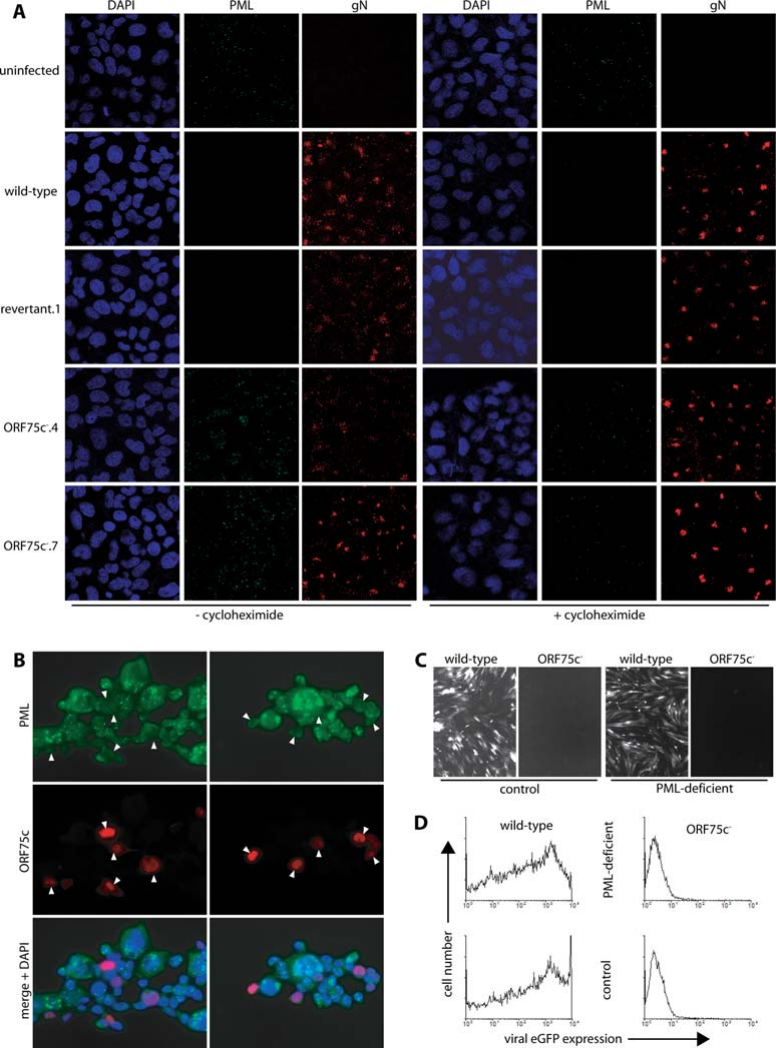
ORF75c disrupts PML bodies. A. HeLa cells were left uninfected or infected with ORF75c^+^ (wild-type, revertant.1) or ORF75c^−^ (ORF75c^−^.4, ORF75c^−^.9) viruses (1000 genomes/cell), with or without 100 µg/ml cycloheximide to block new protein synthesis. 6 h later, the cells were fixed, permeabilized and stained for gN (mAb 3F7, IgG_2a_, red) and PML (mAb PG-M3, IgG_1_, green). Nuclei were counterstained with DAPI (blue). B. 293T cells were transfected with pcDNA3-ORF75c and 48 h later fixed, permeabilized and stained for ORF75c (mAb BN-6C12, IgG_2a_, red) and PML (mAb PG-M3, IgG_1_, green). Nuclei were counterstained with DAPI (blue). Two independent transfections are shown. The arrows show ORF75c^+^ cells. C. Human foreskin fibroblasts made PML-deficient by siRNA expression or expressing a control siRNA were infected (1000 genomes/cell) with wild-type or ORF75c^−^.4 BAC^+^ MuHV-4. Viral infection was assessed 18 h later by viral eGFP expression. D. A similar experiment to C, but with viral eGFP expression quantitated by flow cytometry.

An infection block between genome circularization and viral gene expression suggested that PML bodies (ND10 domains) might be involved in the ORF75c^−^ phenotype. PML bodies constitute a major innate defence against incoming viral genomes, and are a common target for immediate-early herpesvirus gene products [40]. 4 h after exposure to wild-type MuHV-4 virions, HeLa cells showed an almost complete loss of PML bodies (Fig. 6A). This effect was not blocked by cycloheximide, consistent with it being due to a virion component rather than an immediate-early gene product. In contrast to wild-type infection, exposing HeLa cells to ORF75c^−^ virions made their PML bodies more prominent. The increase in PML staining depended on new protein synthesis, as it was blocked by cycloheximide, but even with cycloheximide present ORF75c^−^ virions caused no loss of PML staining. Therefore ORF75c itself or a virion protein functionally dependent on ORF75c disrupted the PML bodies of newly infected cells.

Transfecting expression plasmids provided evidence that PML body disruption is a direct action of ORF75c (Fig. 6B). PML bodies were visible as discrete intra-nuclear dots in ORF75c-negative cells, but were either redistributed to the nuclear margin or not visible at all in 293T cells expressing ORF75c. We tested whether a lack of PML disruption could explain the ORF75c replication deficit by exposing PML-deficient cells to wild-type and ORF75c^−^ virions. A severe ORF75c-dependent block to viral eGFP expression remained (Fig. 6C). Thus, although ORF75c disassembled PML bodies, this action was insufficient to explain the replication deficit of ORF75c^−^ virions.

### ORF75c^−^ virion capsids show defective migration to the nuclear margin

**Figure 7:**
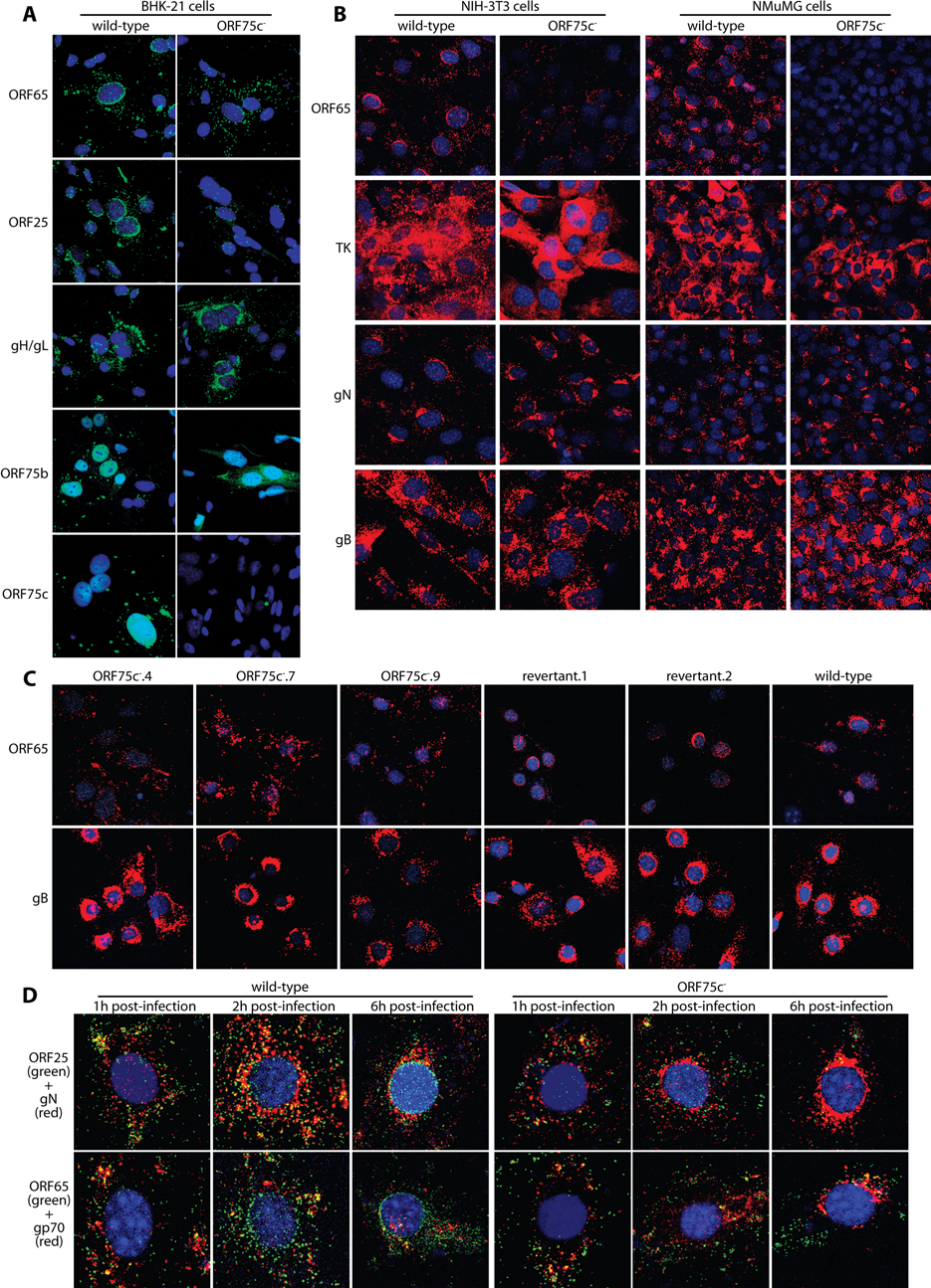
ORF75c disruption impairs the transport of incoming capsids. A. BHK-21 cells were infected (1000 genomes/cell, 6 h) with wild-type or ORF75c^−^.4 MuHV-4, then stained (red) for ORF65 capsid component (mAb MG-12B8), ORF25 capsid component (mAb BH-6D3), gH/gL (mAb T2C12), ORF75b (mAb CS-4A1) or ORF75c (mAb BN-6C12). Nuclei were counter stained with DAPI (blue). B. NIH-3T3 cells or NMuMG cells were infected (1000 genomes/cell, 6 h) with wild-type or ORF75c^−^.4 MuHV-4, then stained (red) for ORF65 (mAb MG-12B8), TK (mAb CS-4A5), gN (mAb 3F7) or gB (mAb MG-4D11). Nuclei were counterstained with DAPI (blue). C. BHK-21 cells were infected (1000 genomes/cell, 6 h) with ORF75c+ (wild type, revertant.1 or revertant.2) or ORF75c^−^ (ORF75c^−^.4, ORF75c^−^.7, ORF75c^−^.9) MuHV-4, then stained for ORF65 or gB as in B. D. NIH-3T3 cells were infected (1000 genomes/cell) with wild-type or ORF75^−^.4 MuHV-4 for 1, 2 or 6 h. The cells were then fixed, permeabilized and co-stained for ORF25 (BH-6D3, IgG_1_, red) and gN (3F7, IgG_2a_, green), or for ORF65 (MG-12B8, IgG_2a_, red) and gp70 (T3B8, IgG_1_, green), using isotype-specific detection. Nuclei were counterstained with DAPI (blue).

The relative increase in circular over linear ORF75c^−^ genomes on Gardella gels of infected cells (Fig. 5B) argued that some ORF75c^−^ genomes reaching the nucleus failed to initiate lytic replication. However, it remained unclear what fraction of input genomes reached the nucleus. For example, any still trapped in capsids might not have been efficiently released by SDS/proteinase K treatment. And although some input genomes could be rescued by super-infection (Fig. 5C), the fraction of eGFP^+^ cells remained low even with an input of approximately 1000 ORF75c^−^ genomes per cell, arguing that the rescue was inefficient. Another block to infection therefore seemed likely. To address where this might be, we examined by immunofluorescence the distribution of capsid antigens in BHK-21 cells 6 h after exposure to ORF75^+^ or ORF75^−^ virions (Fig. 7A). The delivery of gH/gL was similar between viruses; ORF75b also reached the nucleus regardless of ORF75c, consistent with normal membrane fusion; but ORF75c^−^ virions showed a major defect in capsid transport to the nuclear margin. With the wild-type, ORF25 and ORF65 capsid antigens were each clustered around the nuclear margin. With the ORF75c^−^.4 mutant, less capsid staining was evident and it was scattered in the cytoplasm rather than peri-nuclear.

The same was true of NIH-3T3 fibroblast and NMuMG epithelial cell infections (Fig. 7B): incoming gB, gN and TK were similarly distributed 6 h after exposure to ORF75c^+^ or ORF75c^−^ virions, but without ORF75c there was less capsid staining and what staining there was was scattered in the cytoplasm rather than concentrated around the nuclear margin. All the ORF75c mutants showed the same phenotype, while revertant viruses were normal (Fig. 7C). Imaging after 1 h (Fig. 7D) showed little difference in ORF75c^+^ versus ORF75c^−^ capsid staining, but after 2 h there was less accessible capsid antigen for the ORF75c^−^ mutants, and after 6 h the distribution of capsid antigens was clearly different. Thus, the capsids of ORF75c^−^ virions failed to be properly revealed (or were degraded) and failed to migrate to the nuclear margin.

### ORF75c^−^ mutants fail to establish a detectable infection *in vivo*

**Figure 8:**
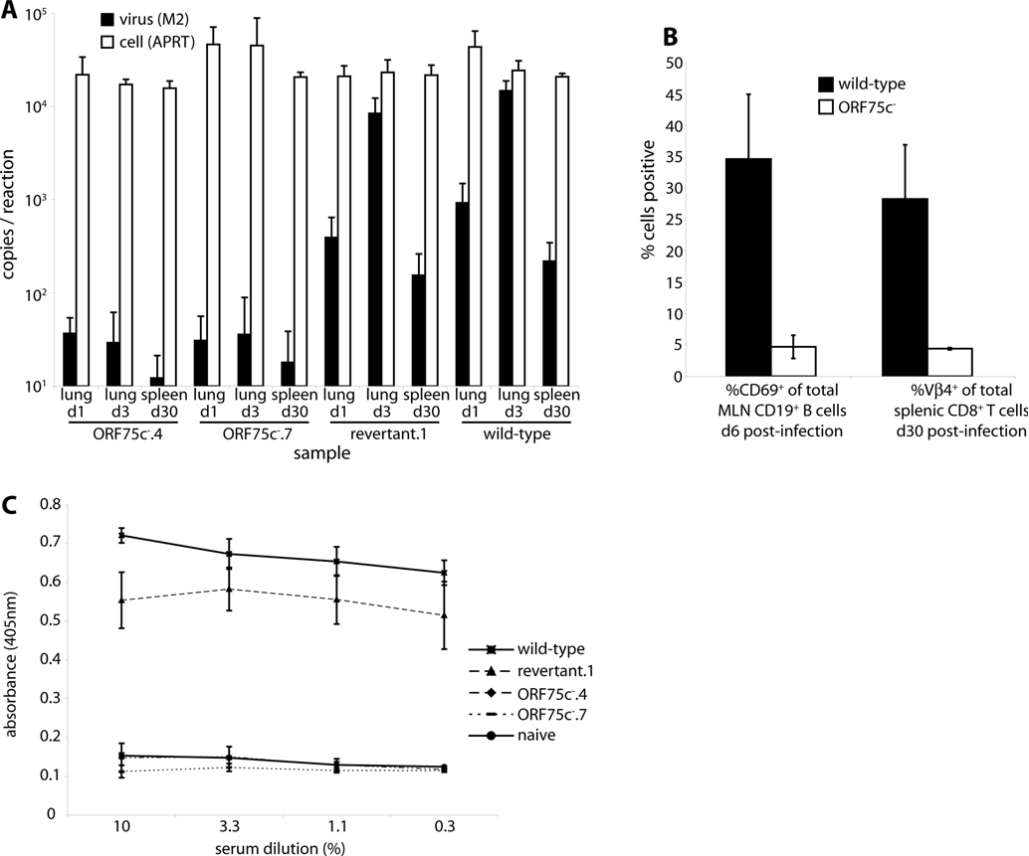
ORF75c disruption impairs host colonization. A. Mice were infected intranasally (10^6^ genomes each, equivalent to 10^3^ p.f.u. for the wild-type) with ORF75^+^ (wild-type, revertant.1) or ORF75^−^ (ORF75c^−^.4, ORF75c^−^.7) viruses. Lungs were collected 1 or 3 days later and spleens 30 days later. Viral genome loads in 100 ng DNA from each sample was measured by real-time PCR of the M2 locus. Part of the adenosine phosphoribosyl transferasegene was amplified as a cellular control. Each bar shows mean and SEM values for groups of 5 mice. B. Mice were infected intranasally (10^6^ genomes each) with wild-type or ORF75c^−^.4 viruses. B cell activation in mediastinal lymph nodes was assayed after 6 d by flow cytometry of the CD69 activation marker on CD19^+^ B cells. Equivalent B cells from naive mice are typically 5%CD69^+^. After 30 d, virus-driven CD8^+^Vβ4^+^ T cell expansion was measured by flow cytometry of spleen cells. Typically 5% of CD8^+^ splenic T cells are Vβ4^+^ in uninfected mice. Each bar shows mean and SEM values for 5 individual mice. C. Mice were infected as in A, then analyzed 30 days later for MuHV-4-specific serum IgG by ELISA.

A key question for any MuHV-4 molecular deficit is how it manifests *in vivo*. We addressed this here by intranasal infection of mice with ORF75c^−^ and ORF75c^+^ viruses (Fig. 8). Since the former were hard to detect by plaque assay, we used real-time PCR of viral genomes to measure host colonization (Fig. 8A). Wild-type genome numbers increased from day 1 to day 3 post-inoculation in the lung and were readily detectable at 30 days post-inoculation in the spleen. ORF75c^−^ genomes were also detectable in the lung after intranasal infection, but failed to increase in number, implying that we were probably detecting just the input virus.

The severe *in vivo* lytic replication of MuHV-4 thymidine kinase knockouts does not stop them colonizing the spleen by 30 days post-inoculation [23]. However, viral genomes were only detectable in the spleen at very low levels 30 days after ORF75c^−^ infection, close to the limit of assay sensitivity (Fig. 8A). There was also no sign of ORF75c^−^ viruses inducing virus-driven B cell activation (Fig. 8B), Vβ4^+^CD8^+^ T cell expansion (Fig. 8B) or virus-specific serum IgG (Fig. 8C). A lack of ORF75c therefore caused a severe defect in host colonization, with little evidence of either lytic replication or normal latency establishment.

## Discussion

DNA sequence alignments tell us the evolutionary histories of viral genes but not necessarily their present functions, as similar protein folds can be adapted to very different purposes. Herpesvirus DNA replication enzymes are generally early gene products, whereas ORFs 75a/b/c all encoded tegument proteins, and none of these FGARAT homologues complemented FGARAT-deficient cells. The functions of herpesvirus tegument proteins in newly infected cells are not well defined. Lytic functions such as DNA replication seem unlikely, as incoming genomes often establish latency. Indeed, the transcriptional activation [35] and host shutoff [41] functions of the Herpes simplex virus tegument may be permitted only because they largely fail to function in neurons. We identified two functions for ORF75c: capsid transport to the nuclear margin and PML body disruption. The dominant defect appeared to be capsid transport, since ORF75c knockouts remained highly attenuated in PML-deficient cells.

Most analyses of herpesvirus capsid transport have focussed on Herpes simplex virus [42] and Pseudorabies virus [43], which travel long distances in neurons during latency establishment and reactivation. Tegument proteins are sequentially released, with the inner tegument proteins presumably interacting with host motor proteins [44]. But what these proteins are and how they function remains unclear [45]. MuHV-4 is latent in B cells, and therefore has less need for long-distance intracellular capsid transport. In the only cell types examined to date - fibroblasts [28], epithelial cells [46] and dendritic cells [36] - infection occurs via endocytosis, and capsids are released from late endosomes close to the nuclear margin. Nevertheless, capsid transport over a short distance may still be an important tegument function. The most direct explanation for the ORF75c^−^ phenotype would therefore be that ORF75c links the viral capsid to host motor proteins for transport to nuclear pores. ORF75c could then be released to enter the nucleus and carry out its second function of PML body disruption.

A second possibility is that the role of ORF75c in capsid transport is indirect. For example, ORF75c could recruit other proteins into the tegument or be required for an orderly release of tegument proteins after membrane fusion. A third possibility is that capsid transport occurs by default, and poor transport reflects the activation of host innate defences that are normally blocked by ORF75c. This was suggested by ORF75c affecting PML, since PML is one of a large family of structurally related tripartite motif (TRIM) proteins, at least some of which participate in anti-viral defence [47]. TRIM functions are largely undefined, but TRIM1 and TRIM5α are known to target incoming retroviruses before nuclear entry. Herpesviruses are equally ancient pathogens, so important interactions with TRIMs would not be a surprise. If ORF75c can interact with one TRIM it could potentially interact with others too, and if ORF75c can interact, so might ORFs 75a and 75b. The multiplicity and diversity of host TRIMs would then provide an explanation for the duplication and diversity of gamma-herpesvirus FGARAT homologues.

In summary, the evolution of a captured host FGARAT into a set of viral tegument proteins had suggested that these proteins no longer function mainly as FGARATs, and such was found to be the case. ORF75c showed functional similarity to the EBV BNRF1, in that infection by the knockout virus was inhibited at a pre-nuclear entry step. ORFs 75a and 75b, although related in DNA sequence to ORF75c and also present in the tegument, were unable to substitute for it, indicating that their functions are distinct. The molecular explanation for the ORF75c^−^ phenotype of defective incoming capsid transport remains incomplete. However, the effect of ORF75c on PML (TRIM19), while insufficient by itself to explain the low infectivity of ORF75c^−^ mutants, suggested that ORF75c might also target other TRIMs. Innate anti-viral defences are plausible targets for the herpesvirus tegument, as incoming genomes must reach the nucleus silently enough for latently infected cells not to become immune targets. Mapping the ORF75 host interaction partners may tell us how this is achieved.

## Supporting Information

Figure S1 (2.81 MB TIF)
