# Supplementary figures and images for: Multiple Functions for ORF75c in Murid Herpesvirus-4 Infection
Source: PLoS One. 2008 Jul 23;3(7):e2781. doi: 10.1371/journal.pone.0002781 (PMC2464709; doi:10.1371/journal.pone.0002781)

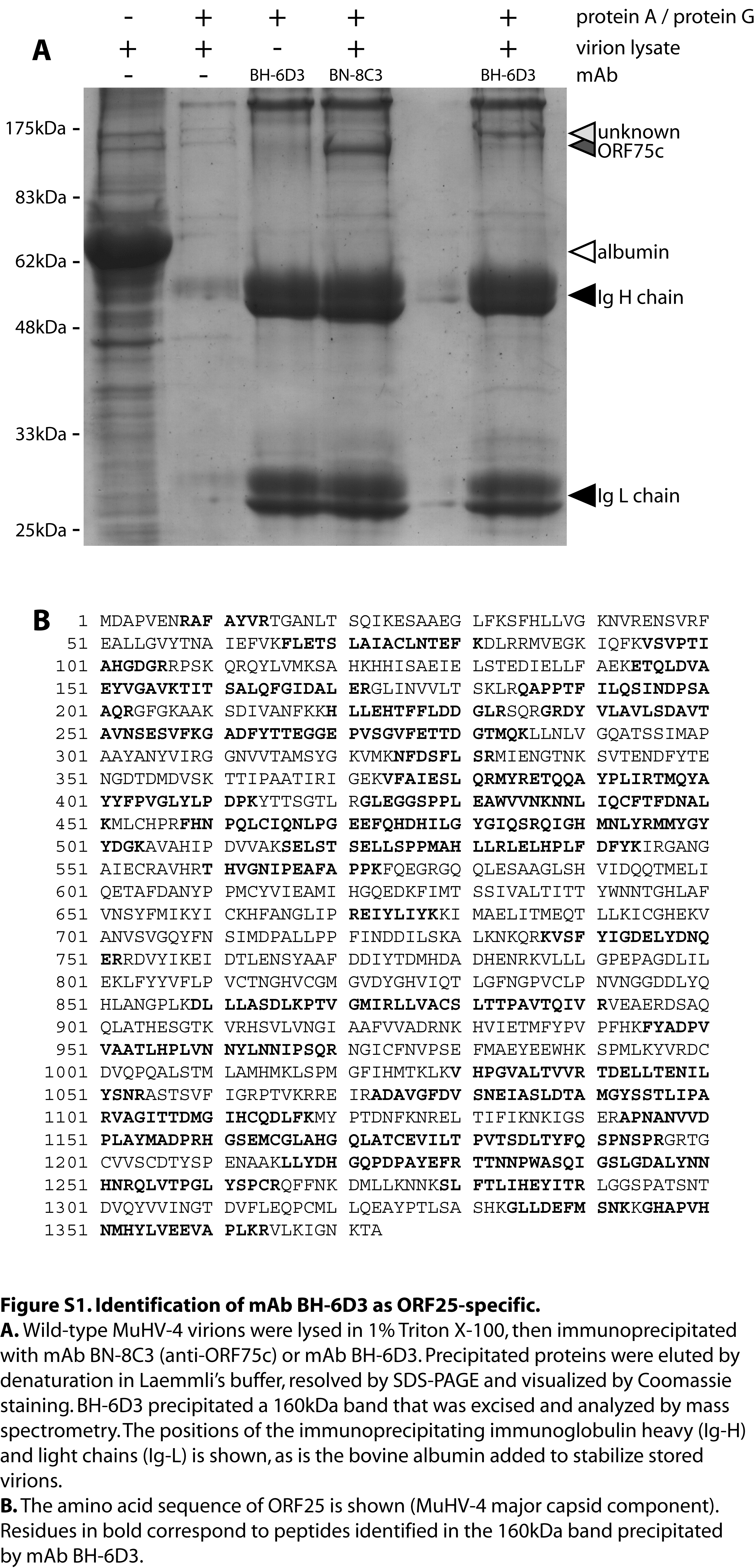

Supplement: Figure S1 — (2.81 MB TIF) [file pone.0002781.s001.tif]
